# Transcriptional and Metabolic Changes Associated with Phytoglobin Expression during Germination of Barley Seeds

**DOI:** 10.3390/ijms21082796

**Published:** 2020-04-17

**Authors:** Somaieh Zafari, Kim H. Hebelstrup, Abir U. Igamberdiev

**Affiliations:** 1 Department of Biology, Memorial University of Newfoundland, St. John’s, NL A1B 3X9, Canada; szafari@mun.ca; 2 Department of Molecular Biology and Genetics, Aarhus University, Flakkebjerg, DK-4200 Slagelse, Denmark; kim.hebelstrup@mbg.au.dk

**Keywords:** ATP/ADP ratio, fermentation, mitochondria, nitric oxide, phytoglobin, seed germination

## Abstract

To understand how the class 1 phytoglobin is involved in germination process via the modulation of the nitric oxide (NO) metabolism, we performed the analysis of physiological and molecular parameters in the embryos of transgenic barley (*Hordeum vulgare* L. cv Golden Promise) plants differing in expression levels of the phytoglobin (*Pgb1*) gene during the first 48 h of germination. Overexpression of *Pgb1* resulted in a higher rate of germination, higher protein content and higher ATP/ADP ratios. This was accompanied by a lower rate of NO emission after radicle protrusion, as compared to the wild type and downregulating line, and a lower rate of *S*-nitrosylation of proteins in the first hours postimbibition. The rate of fermentation estimated by the expression and activity of alcohol dehydrogenase was significantly higher in the *Pgb1* downregulating line, the same tendency was observed for nitrate reductase expression. The genes encoding succinate dehydrogenase and pyruvate dehydrogenase complex subunits were more actively expressed in embryos of the seeds overexpressing *Pgb1*. It is concluded that *Pgb1* expression in embryo is essential for the maintenance of redox and energy balance before radicle protrusion, when seeds experience low internal oxygen concentration and exerts the effect on metabolism during the initial development of seedlings.

## 1. Introduction

Seed germination represents an initial and critical phase of the life cycle in plants. It starts with water uptake, resulting in a transition from the quiescent state of metabolism in the dry seed to the high metabolic activity upon hydration [1], and finishes with radicle protrusion, which is an observable indicator of the completion of germination [2]. After imbibition, seeds develop highly hypoxic conditions limiting mitochondrial respiration [3], which results in the increase of the reduction level of electron transferring components, triggering the generation of reactive species of oxygen (ROS) and of nitrogen (RNS) [4,5]. Under low oxygen conditions, plants have a limited capacity to substitute oxygen with nitrite as the terminal electron acceptor [6]. This nitrite can be reduced to nitric oxide (NO) by various iron-containing proteins and molybdocofactors. The anoxically induced phytoglobin (Pgb) converts this NO to nitrate, which is metabolized back into nitrite via nitrate reductase (NR) for the continuation of NO production [7]. The cycle of the nitrate- and nitrite-driven redox reactions is defined as the phytoglobin-nitric oxide (Pgb-NO) cycle, playing a key role in the maintenance of the energy status of the embryo under hypoxic conditions [8]. Besides NO oxygenation by Pgb, denitrosylation of *S*-nitrosoglutathione (GSNO) catalyzed by *S*-nitrosoglutathione reductase (GSNOR) is another way to metabolize NO. GSNO is the storage and transport form for NO in seeds [9], which has a crucial impact on seed germination [10].

Germination has a high demand of energy, which is fulfilled due to the functional mitochondria that remain stable and efficient through the assembly of the mitochondrial protein complexes during imbibition [11,12]. Once seeds are imbibed, the tricarboxylic acid (TCA) cycle supplies intermediates and energy to support seed germination and seedling growth. This cycle couples with oxidative phosphorylation to produce ATP [13]. The mobilization of starch from the endosperm would seemingly proceed through glycolysis and the TCA cycle. The TCA cycle cannot function without the acetyl-CoA input produced by pyruvate dehydrogenase complex (PDC). Succinate dehydrogenase (SDH) activity in the scutellum of germinating cereal seeds reflects the need for succinate conversion for the continuous operation of the TCA cycle, and for the utilization of succinate produced in the glyoxylate cycle [14]. NO inhibits mitochondrial respiration by reversible binding to cytochrome c oxidase [15], while it does not affect the alternative oxidase (AOX) [15]. NO action on the mitochondrial electron transport affects oxidative phosphorylation [16]. An interplay and flexible equilibrium between ROS, NO, and mitochondrial respiration is needed for the maintenance of energy status within the seed in the course of germination.

Plants possess non-energy conserving electron transport pathways in mitochondria, which couple the oxidation of NADH and NADPH with the reduction of O_2_ to H_2_O without generating a proton motive force [17]. These pathways include the type II NAD(P)H dehydrogenases (NDs) on both sides of the inner membrane of the mitochondria and AOX. AOX and NDs are encoded by multigene families. In barley, AOX is encoded by four genes: *HvAOX1a*, *HvAOX1c*, *HvAOX1d1* and *HvAOX1d2*. All three subfamilies of plant ND genes, *NDA*, *NDB* and *NDC*, were identified in barley [18]. In this study, we analyzed *AOX1a* and *AOX1d1*, and *NDB2* and *NDB3* encoding NDB proteins oxidizing NADH and facing the outer side of the inner membrane. Non-energy conserving electron transport, which includes rotenone-insensitive NADH and NADPH dehydrogenases and AOX, represents a tool for relaxing the coupling of the respiratory carbon oxidation pathways, electron transport, and ATP turnover, thus establishing metabolic homeostasis during germination [18,19]. The involvement of these pathways was studied in plants mostly in relation to photosynthesis, while their role of germinating seeds is also important [20]. It may be connected with NO metabolism and operation of the Pgb-NO cycle [21], providing the possibility of oxidation of the cytosolic NADH and the prevention of ROS and RNS formation.

The apparent imbalance between the respiration levels (measured as oxygen uptake) of cereal species and the relative abundance of mitochondrial ATP suggests that energy charge may be supplied by alternative pathways (e.g., fermentation) during seed germination [22]. This is in accordance with a drastic increase in the ATP level in the first hours of imbibition [23]. Alcoholic fermentation is induced during germination of rice seeds to provide energy when oxygen is deficient for providing normal respiration [24]. The energy obtained via fermentation facilitates seed germination and radicles protrusion to overcome anaerobic stress. However, there is an alternative to the classic fermentation pathways [6], associated with the turnover of NO in the Pgb-NO cycle [25]. This cycle can operate at the concentrations of oxygen two orders of magnitude lower than required to support the oxygenic respiration and oxidize NADH and produce ATP at the intensities comparable to or exceeding the glycolytic levels [8].

While the role of NO in seed biology has been studied extensively, the information about the function of phytoglobin in NO interactions with other molecules involved in fermentation, TCA, and electron transport, including the alternative pathway, remains scarce. This work clarifies how the changes in the endogenous level of phytoglobin affect the underlying molecular features to control the onset of germination and manage the energy crisis, which dictate the tolerance to anoxic step of germination, and finally support the growth and development of embryonic axis.

## 2. Results

**Figure 1:**
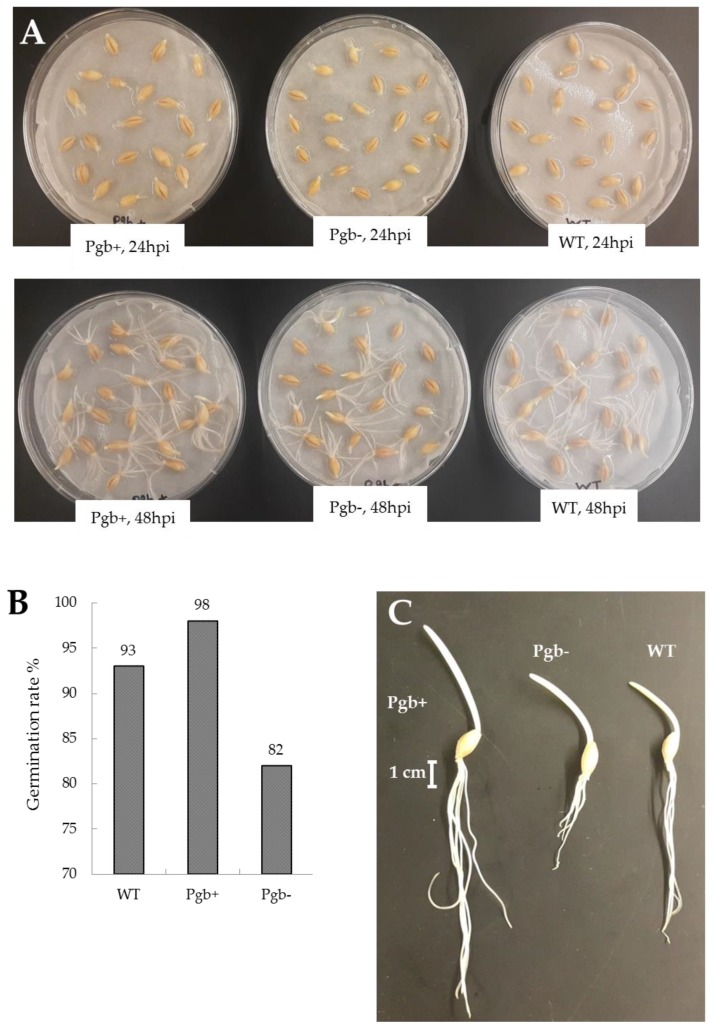
Germination of barley seeds differentially expressing class 1 phytoglobin (*Pgb1*). (**A**) Image of germinating seeds of Golden Promise cultivar of barley with overexpressed (Pgb+) and knockdown (Pgb-) phytoglobin compared to wild type (WT) at 24 and 48 h post imbibition. (**B**) Germination rate of barley seeds differentially expressing *Pgb1*. The typical result of the experiment with 250 seeds. (**C**) Image of 3-day-old barley seedlings with overexpressed (Pgb+) and knockdown (Pgb-) phytoglobin compared to wild type (WT).

In this paper, we report the results of a wide-range study of the development of barley embryos differing in expression levels of the *Pgb* gene (Pgb+ and Pgb–) during the first 2 days of germination. Figure 1A presents the images of germination of transgenic barley seeds, as observed at 24 h after imbibition (after radicle protrusion) and at 48 h. Radicle protrusion occurs mostly between 15 and 20 h postimbibition in all lines but the Pgb+ seeds develop a longer radicle by 24 h, as compared to WT and Pgb-.

By 48 h, the root system of the Pgb+ becomes more developed than that of WT and Pgb-. Germination rate of Pgb+ seeds (98%) was significantly higher than of WT seeds (93%) and then Pgb- (82%) (Figure 1B). After 3 days of germination, the Pgb+ seedling exhibited twice as long roots and 1.5 times longer shoots compared to Pgb-, while the WT seedling exerted the intermediate length of roots and shoots (Figure 1C).

**Figure 2:**
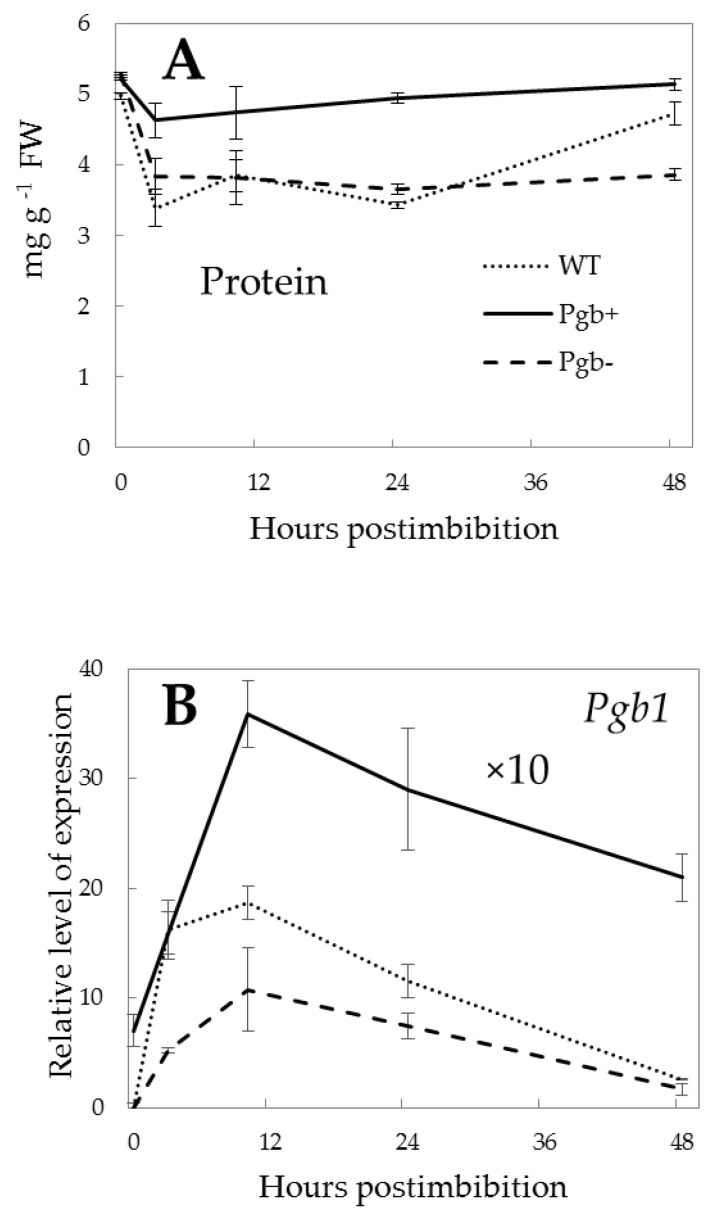
Changes in total protein content (**A**) and *Pgb1* expression (**B**) in the embryo of barley during germination. Solid line: Pgb+, overexpressed phytoglobin; dashed line: Pgb-, knockdown phytoglobin; dotted line: WT, wild type. The values for relative level of expression of Pgb+ should be multiplied by 10 times. The vertical bars represent the values of standard deviations.

The total protein content in the embryo started to decrease immediately after imbibition during the first 3 h (Figure 2A). The decrease was strong (by ~30%) in the WT and Pgb- seeds, while in Pgb+ seeds it was about 10% in the first three hours, and then showed the tendency to increase to the initial level at 48 h. In Pgb- seeds, the total protein remained at lower level, while in the WT it started to increase after 24 h, and reached almost the level as in dry seeds at 48 h. The expression of the *Pgb1* gene sharply increased during 10 h postimbibition (Figure 2B) from zero levels in the dry seeds of WT and Pgb-, and from well detectable levels in Pgb+. At 10 h, the level of expression of *Pgb1* was half in Pgb-, as compared to the WT, and about 20 times higher in Pgb+ seeds. At 48 h postimbibition, the level of *Pgb1* mRNA was quite low in the Pgb- and WT lines but remained high in Pgb+.

**Figure 3:**
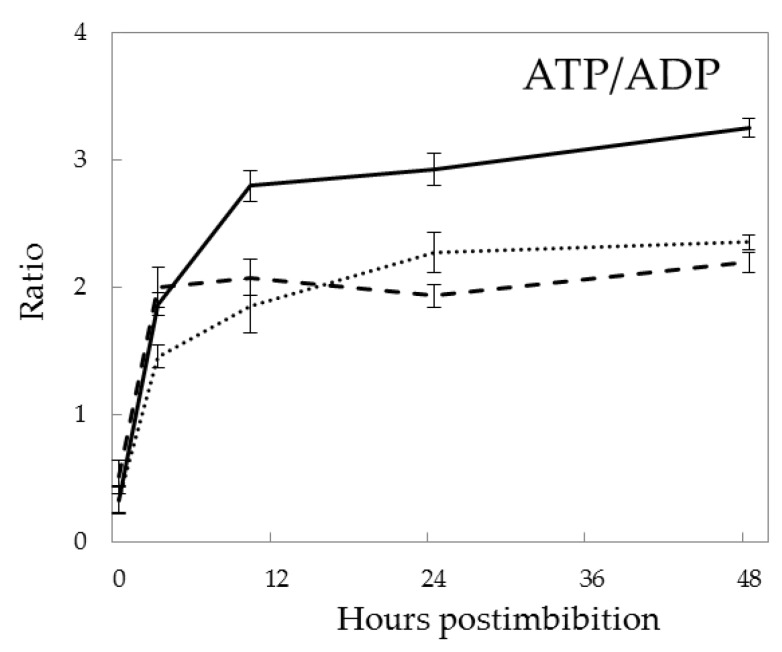
ATP/ADP ratio in barley embryo during germination depending on expression of *Pgb1*. The symbols are the same as in Figure 2.

The ATP/ADP ratio in the embryo strongly increased in the first 3 h after imbibition, then maintained unchanged in Pgb- embryos and gradually but slightly increased in the WT (Figure 3). In Pgb+ embryos, the ATP/ADP ratio increased more significantly and remained ~1.5 times higher than in Pgb- and WT embryos at 24–48 h.

**Figure 4:**
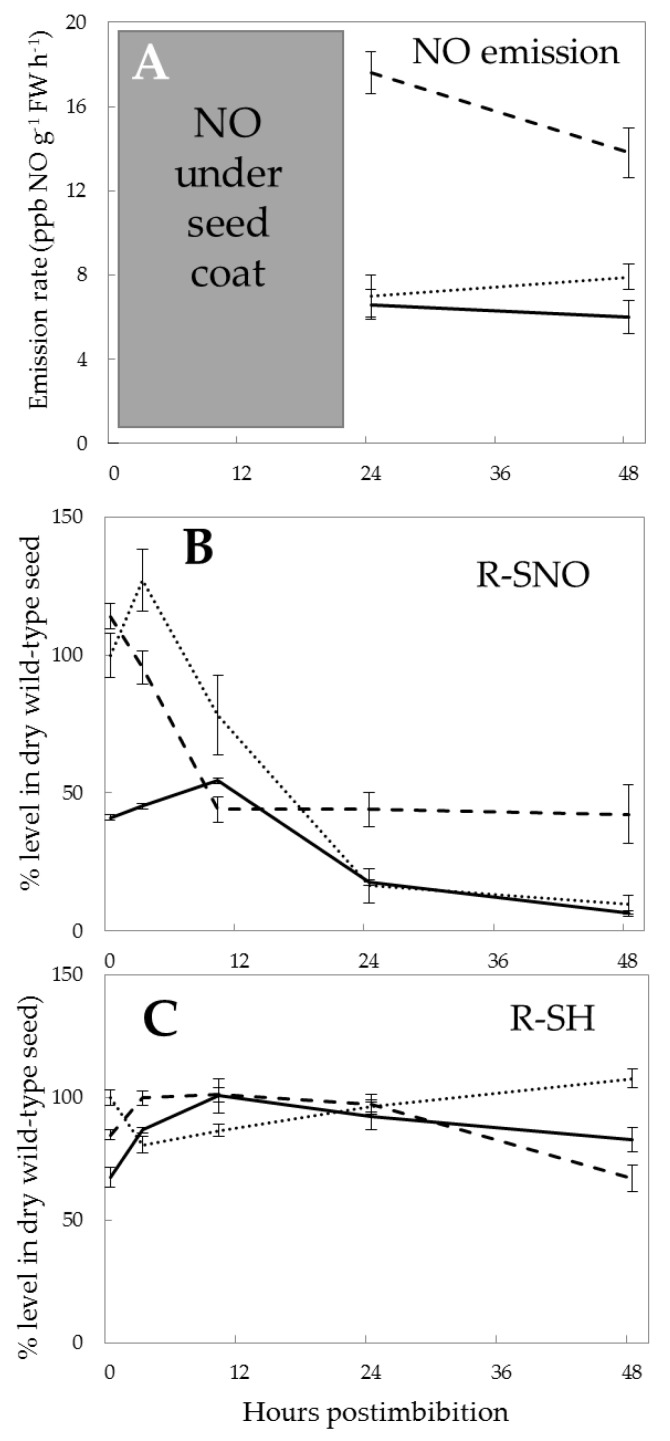
Nitric oxide (NO) emissions (**A**), changes in the quantity of nitrosylated (−SNO) groups in proteins (**B**), and the quantity of sulfhydryl groups in proteins (**C**) in the embryos of barley seeds differentially expressing *Pgb1* during germination. NO emission was recorded by chemiluminescent method as described in Methods; detection was possible after breakage of the seed coat (radicle protrusion). The symbols are the same as in Figure 2.

It was possible to detect NO by the chemiluminescent method only after radicle protrusion (Figure 4A). The rate of NO emission was more than twice as high in Pgb- than in Pgb+ and the WT at 24 h, and remained almost at the same rate at 48 h. It was possible to detect a statistically significant difference between the WT and Pgb+, in which NO production was the lowest (Figure 4A). Although we could not detect NO emissions before radicle protrusion by the applied method, the rate of *S*-nitrosylation of proteins (Figure 4B) was less than half in Pgb+ than in the Pgb- and WT in dry seeds at 3 h of germination. At 10 h, the levels were not quite different between the lines, except a slightly higher level in the WT, while later, at 24 and 48 h, Pgb- exhibited the same level of nitrosothiols as at 10 h, and the nitrosothiol level in the Pgb+ and WT gradually decreased to very low values at 48 h. While the level of nitrosothiols essentially differed depending on *Pgb1* expression, the concentration of SH-groups showed essential but smaller differences between the lines in the first hours and after two days postimbibition (Figure 4C). The reduced concentration of nitrosylated (−SNO) groups in the proteins of dry seeds corresponding to the overexpressing line can be related to the capacity of scavenging NO even in dry seeds, when *Pgb1* exhibited a certain level of expression in the overexpressing line and was practically absent in the wild type and downregulating line (Figure 2). Before the decreasing trend, the increase of nitrosylation in the WT embryos was accompanied by the opposite trend of free SH-groups in proteins. In the Pgb+ embryos, the level of R-SNO, being markedly reduced after 10 h from imbibition, did not show a correlation with the level of RSH, which exhibited no significant changes.

**Figure 5:**
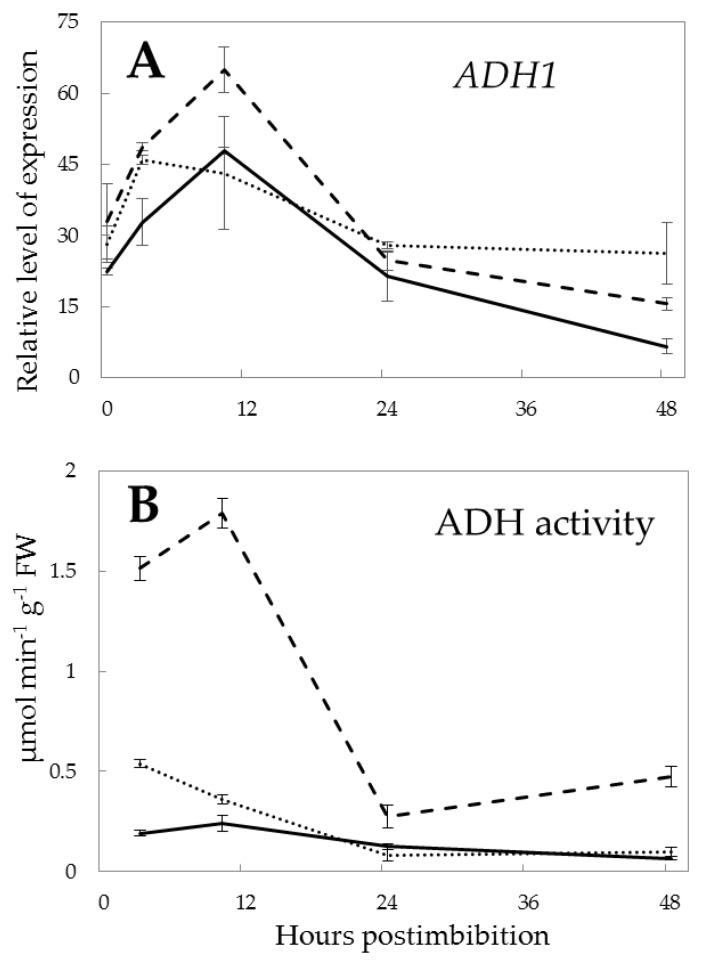
Expression of alcohol dehydrogenase (*ADH1)* (**A**) and its activity (**B**) in the embryos of barley seeds differentially expressing *Pgb1* during germination. The symbols are the same as in Figure 2.

The rate of fermentation estimated by the expression and activity of alcohol dehydrogenase (ADH) was strongly dependent on the *Pgb1* expression. The expression of *ADH1* was lower almost by 1.3 times in Pgb+ seeds than in the Pgb- and WT seeds in the first 3 h after imbibition. It was almost the same in all lines at 24 h but became lower again in Pgb+ after 48 h (Figure 5A). The activity of ADH was higher in Pgb- embryo by more than seven times than in the Pgb+, and by five times than in the WT before radicle protrusion, in the first 10 h after imbibition (Figure 5B).

**Figure 6:**
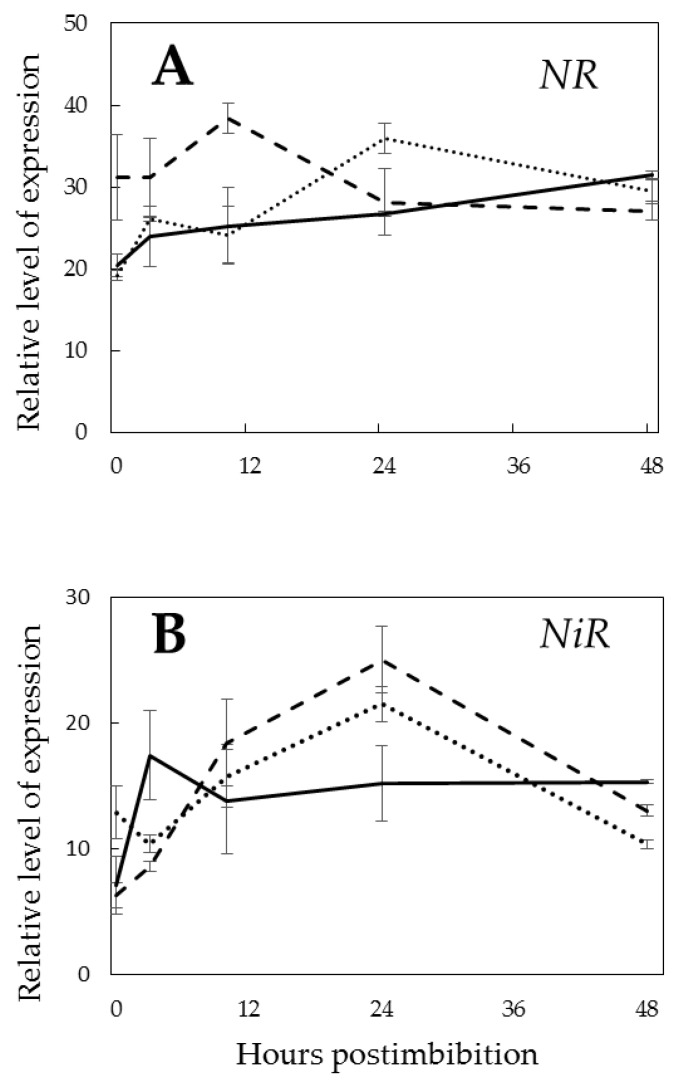
Expression of nitrate reductase (*NR*) (**A**) and nitrite reductase (*NiR*) (**B**) in the embryos of barley seeds differentially expressing *Pgb1* during germination. The symbols are the same as in Figure 2.

The expression profiles of nitrate reductase (*NR*) and nitrite reductase (*NiR*) genes revealed a higher expression of *NR* in the Pgb- line before radicle protrusion, with the highest difference measured at 10 h postimbibition (Figure 6A). The profile of *NiR* expression was almost flat in Pgb+, while in the Pgb- and WT, the expression increased by 24 h being higher at that point than in the Pgb+ and then decreased (Figure 6B).

**Figure 7:**
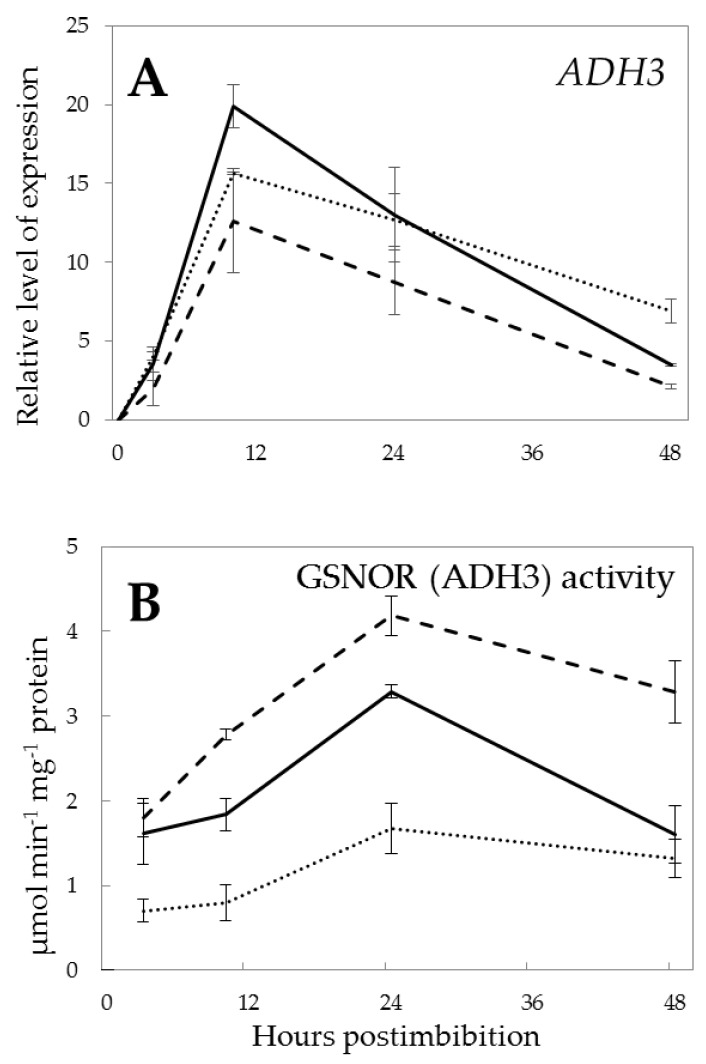
Expression of *ADH3* (**A**) and GSNO reductase activity (**B**) in the embryos of barley seeds differentially expressing *Pgb1* during germination. The symbols are the same as in Figure 2.

The expression and activity of GSNOR followed a similar trend in all three types of the barley embryos (Figure 7). However, the expression of *GSNOR* in Pgb+ embryos was slightly higher than that of the Pgb- embryos at 10 h from imbibition (Figure 7A), while the highest activity was observed on the Pgb- embryos (Figure 7B). Generally, the RNS-scavenging activity of GSNOR increased on the first day of seed germination, in concert with the decreasing trend of protein *S*-nitrosylation (Figure 4B).

**Figure 8:**
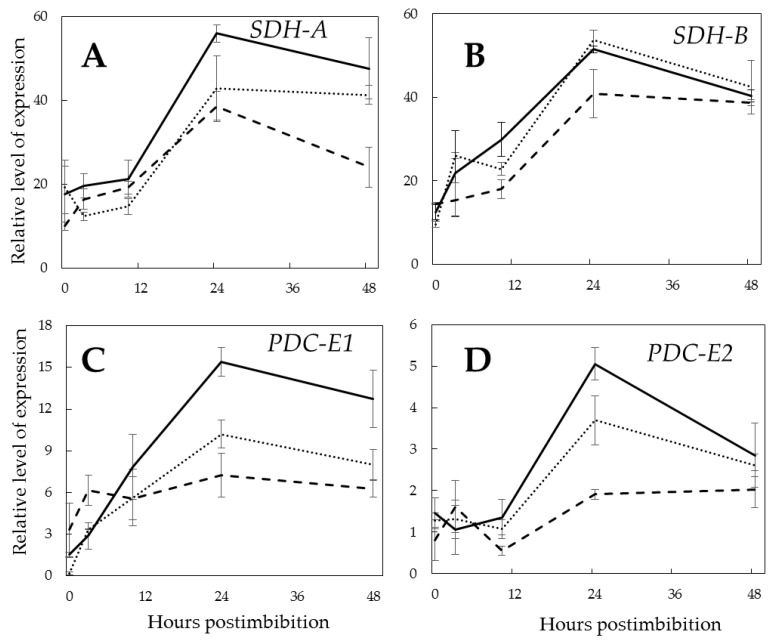
Expression of the genes encoding succinate dehydrogenase subunit A (*SDH-A*) (**A**) and subunit B (*SDH-B*) (**B**), and pyruvate dehydrogenase complex components E1 (*PDC-E1*) (**C**), and E2 (*PDC-E2*) (**D**) in the embryos of barley seeds differentially expressing *Pgb1* during germination. The symbols are the same as in Figure 2.

We have studied the expression of genes encoding SDH subunits A (flavoprotein subunit) and B (iron–sulfur protein subunit) (Figure 8A,B) and PDC subunits E1 (pyruvate dehydrogenase) and E2 (dihydrolipoyl acetyl-transferase) (Figure 8C,D). Generally, all these genes were upregulated in all types of embryos after radicle protrusion when the aerobic metabolism becomes more intensive, and reached higher values in the Pgb+ embryos, although the differences in *SDH-B* and *PDC-E2* were less pronounced.

**Figure 9:**
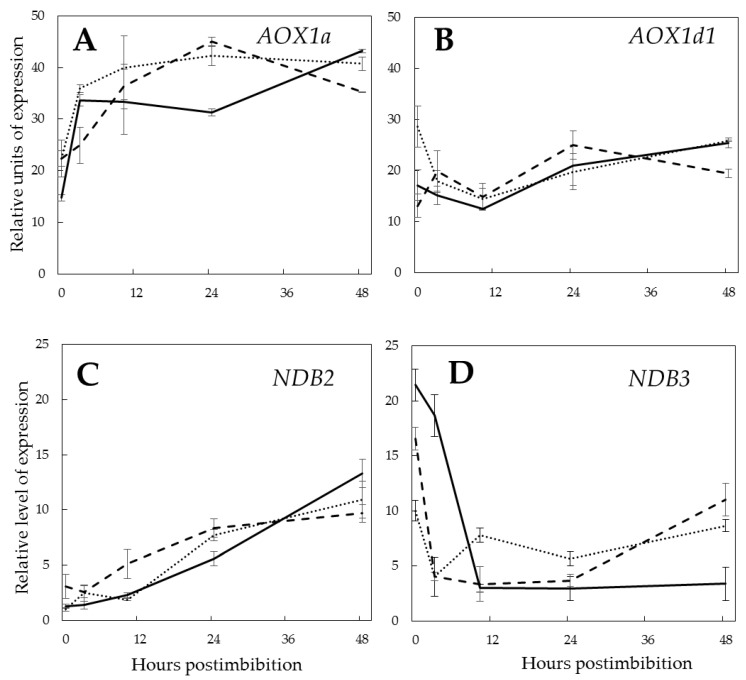
Expression of alternative oxidase genes *AOX1a* (**A**) and *AOX1d1* (**B**) and of external NADH dehydrogenase genes *NDB2* (**C**) and *NDB3* (**D**) in the embryos of barley seeds differentially expressing *Pgb1* during germination. The symbols are the same as in Figure 2.

Expression of the genes encoding the enzymatic members of the non-coupled respiratory pathways, alternative oxidase (*AOX1a* and *AOX1d1*) and external NADH dehydrogenases (*NDB2* and *NDB3*) revealed a specific pattern in the course of germination (Figure 9A–D). The transcripts of *AOX1a* increased in the first 3 h of germination, with the slower increasing trend in Pgb- embryos. Expression of the gene encoding *AOX1d1* was lower and did not show correlation with *Pgb1* expression. The expression of *NDB2* gradually increased during germination with no correlation with *Pgb1*, while the expression of *NDB3* in the dry and 3 h imbibed Pgb+ seeds was higher, then started to decrease during germination, and by 48 h reached s much lower level than in the WT and Pgb- lines.

## 3. Discussion

### 3.1. Anaerobic Conditions in Germinating Seeds and Expression Of Class 1 Phytoglobin

Seed germination is a complex process that begins by imbibition and leads to anatomical structure protrusion initiating seedling development. During germination, rapid oxygen depletion makes the environment inside the seed close to anaerobic [26]. After radicle protrusion, oxygen concentrations return gradually to aerobic, resulting in the active mobilization of storage reserves, followed by seedling development. Based on the previous estimations [26], we assume that by 3–5 h from imbibition, most of oxygen is depleted, leading to mostly anaerobic conditions until the protrusion of a radicle. In our study, the first radicles appeared between 15 and 20 h, and by 24 h, all the seeds developed radicles (Figure 1A).

Anaerobic conditions are characterized by the development of fermentation [27], and by the conversion of nitrite to NO [21]. NO is metabolized by the induced Pgb1 to nitrate, the latter is reduced to nitrite by NR [21]. The sequence of reactions called the phytoglobin-nitric oxide (Pgb-NO) cycle [6] operates as a substitute to classic fermentation pathways at low oxygen, and is efficient in keeping the redox level under control, as well as in generation of limited quantities of ATP [8]. Expression of *Pgb1* under the hypoxic conditions developed in seeds is an important prerequisite of their successful development. The germination rate of the seeds with *Pgb1* knockdown is decreased as compared to the wild type, and especially to the seeds overexpressing *Pgb1* (Figure 1B). The seedlings from Pgb+ seeds are characterized by a better growth with longer roots and shoots (Figure 1C). This means that the expression of *Pgb1* and efficient NO turnover are important for metabolism of seeds during germination, which is confirmed by the recently published metabolomics and proteomic data obtained on 8-day old seedlings of barley [28]. It was suggested that NO scavenging by Pgb activates transcription factors that are regulated by levels of O_2_ and NO in the N-end rule pathway [28], which is an evolutionarily conserved pathway for protein degradation [29]. It relates the regulation of the in vivo half-life of a protein to the composition of its N-terminal residue [29].

Germination is characterized by the continuing utilization of stored proteins that are used as an important source of amino acids, and for energy production [30]. The protein level decreased in the first three hours postimbibition (Figure 2A), and the drop was minimal in the Pgb+ seeds, indicating that they have an energy source independent on protein utilization. The level of expression of *Pgb1* increases sharply upon imbibition (Figure 2B), reaching the maximum close to 10 h, exhibiting the differences depending on *Pgb1* expression in the lines.

### 3.2. Energy Production During Seed Germination and the Role of NO

A sharp increase in the ATP/ADP ratio was detected at 3 h post imbibition (Figure 3), despite of the depletion of oxygen during this period. The overexpression of *Pgb1* resulted in further increase of ATP/ADP ratio which was stabilized in the Pgb- embryos at a lower level, similarly to the wild type. This indicates that, even under highly anoxic conditions, there exist the pathways promoting ATP synthesis, and *Pgb1* expression is important for its buildup and maintenance.

The main role of Pgb1 is determined to be NO scavenging [6,31] which is confirmed in this study by measuring NO emissions from germinating seeds after radicle protrusion (Figure 4A). Although this method does not make it possible to measure NO before radicle protrusion, as it does not leave seeds protected by seed coat, and the hemoglobin method applied in the previous study [5] is not sufficiently precise, so we can assume high NO concentrations in the first hours postimbibition from *S*-nitrosylation profiles (Figure 4B). From the graph, it is evident that Pgb1 protects from nitrosylation already in the first hours of imbibition. At 10 h, the difference in the Pgb+ line disappears or becomes less pronounced, which means that other mechanisms can be important, including GSNO reductase and other scavenging pathways.

The contribution of Pgb1 to a buildup of ATP [7,8] is apparent from the data on ATP/ADP ratio (Figure 3), indicating that Pgb1 operation considerably supports the physiological performance of the germinating seed. Previous studies showed that *Pgb1* gene expression during hypoxia has proven important for improving energy status in maize cell culture and alfalfa roots [32,33]. The production of NO at the initial stages of germination, when the seeds develop anoxic conditions, was reported earlier [5]. It results in the nitrosylation of SH-groups in peptides such as glutathione, many proteins, free cysteine and its derivatives, the processes controlled by the fine balance of NO manufacturing and scavenging mechanisms [3].

### 3.3. Fermentation and the Pgb-NO Cycle

The development of anaerobic conditions in seeds leads to the induction of fermentation within the first hours post imbibition. Our previous work showed that the higher ATP/ADP ratio is characterized by higher activities of fermentation enzymes (ADH and LDH) in the first hours of the germination of barley seeds [5]. It was also established that the highest activities of fermentation enzymes are observed in the plants downregulating *Pgb1* [34]. We observed the anoxia-triggered increase of *ADH1* expression and activity in barley embryos (Figure 5), confirming that fermentation does play a significant role at the stage when plants rely on the seed’s energy stores. In accordance with our results, the upregulation of fermentation-related genes in barley and Arabidopsis seeds was reported during early imbibition [35,36].

A higher expression of *ADH1* and a several-fold higher ADH activity in the *Pgb1* knockdown line points on the higher use of fermentation for producing energy in the absence of Pgb. In this case, Pgb- embryos attempt to use the fermentation pathway to a higher extent than the Pgb+ and wild type embryos. This supports the statement that NO turnover in the Pgb-NO cycle represents an alternative to fermentation pathways [6,25,37]. Both metabolic processes operate during seed germination, and the reported dependence of *Pgb* expression on the hypoxic conditions indicates the deficiency of oxygen in germinating seed before radicle protrusion [6].

The Pgb-NO cycle, which is based on the turnover of NO, nitrate and nitrite, is essential for controlling O_2_ homeostasis and for supporting low redox and a high energy level. The Pgb-NO cycle relies on the participation of electron transport complexes using nitrite, Pgb, and NR. *NR* expression before radicle protrusion was higher in the *Pgb1* knockdown line, which may be considered as a compensation for lower nitrate production in these seeds from the reaction of NO with Pgb (Figure 6A). Contrarily, expression of *NiR* in the first hours postimbibition was higher in the Pgb+ embryos (Figure 6B). This may indicate that nitrite is utilized not only for NO production, but also for the buildup of ammonia for the synthesis of amino acids, which remains high in anoxia and increases upon NO production [38].

### 3.4. S-Nitrosoglutathione Reductase

While the Pgb-dependent NO scavenging can efficiently suppress the rate of nitrosylation, GSNO, which represents the major pool of nitrosylated compounds [39], is metabolized via GSNOR. Our data demonstrate that GSNOR exhibits the dependence on *Pgb1* expression (Figure 7A). The induction of GSNOR activity in Pgb- seeds may be linked to the necessity of controlling GSNO levels in this line. The more pronounced increase of GSNOR activity in early hours of germination in Pgb- embryos could be attributed to a compensational strategy for the insufficient presence of phytoglobin to modulate NO. Its higher activity, despite lower expression in the Pgb- plants, may be due to the post-translational mechanisms of activation, or higher level of translation of existing mRNAs, although our data do not provide evidence for concrete mechanisms. GSNOR controls the intracellular levels of *S*-nitrosylated proteins and, in turn, it is post-translationally regulated by *S*-nitrosylation, and this modification has been suggested to regulate allosterically the enzyme activity [40,41]. Thus, NO concentration, depending on the balance between pgb level, GSNOR and NR activity, determines the extent of *S*-nitrosylation inside the cell. A low level of nitrosylated proteins on the second day of germination (Figure 4B) can be in accordance with a higher activity of GSNOR and still high expression of *Pgb1*.

### 3.5. Pgb1 and Operation of the TCA Cycle

The TCA cycle is a vital metabolic pathway supplying intermediates and energy for the maintenance of seed germination and seedling growth [42]. SDH catalyzing the conversion of succinate to fumarate is the only enzyme joining both the TCA cycle and ETC [43]. It also utilizes succinate formed in the glyoxylate cycle [44]. The mitochondrial PDC, representing an assembly of three respiratory enzymes, links glycolysis and the TCA cycle. Besides being the entry to TCA cycle, PDC supplies the C intermediate (acetyl-CoA) and NADH for the anabolic processes during seedling development [45].

The high expression of *SDH* and *PDC* in embryos, where *Pgb1* is upregulated, on the second day of germination is in accordance with their enhanced mitochondrial respiratory activity (Figure 8A–D). The studies showed that NO is involved in the inhibition of SDH [46], possibly via nitrosylation of protein thiols and removing iron from the iron-sulfur centers [47]. NO inhibits the upregulation of *SDH* and *PDC* in Pgb- embryos on the second day of germination, when the high metabolic demands are met by aerobic respiration, while Pgb, by scavenging NO, protects SDH from inactivation. Thus, the importance of the Pgb-NO cycle is evident not only at the anaerobic stage, but also after radicle protrusion, when the TCA cycle operation becomes more active. It may prevent the suppression of cytochrome oxidase by NO, which is considered an important mechanism of the regulation of respiratory metabolism [37].

### 3.6. Pgb and the Non-Coupled Respiration

The capacity of seeds to germinate might be related to the regulation of ROS levels generated in the mitochondrial consumption of oxygen, and to the detoxification of the products of fermentation [24,48]. The non-coupled pathways of the mitochondrial electron transport that include AOX and rotenone-insensitive NAD(P)H dehydrogenases, decrease the reduction level of NAD(P)H and ubiquinone, and prevent excessive ROS and RNS formation [49]. Among the NAD(P)H dehydrogenases, the NDB-type enzymes oxidize NADPH (NDB1) and NADH (NDB2 and NDB3) from the outer side of the inner mitochondrial membrane, and can be involved in the oxidation of glycolytic NADH and redox equivalents formed in other cytoplasmic processes. They can participate in seed germination and seedling development by controlling ROS homeostasis during the germination process, and by promoting the cellular redox balance during post-germination development [18,19,50]. Participation of the NDB-type dehydrogenases in NAD(P)H-dependent scavenging of NO via its reaction with superoxide anion shows their role in NO homeostasis [51].

The expression profiles of *AOX1a* and *AOX1d1*, which differ in the regulation by oxo-acids [52], show differences upon germination, indicating that *AOX1a* is inducible upon imbibition, while *AOX1d1* is not. However, both forms, as well as *NDB2*, do not show any significant dependence on expression of *Pgb1*. However, *NDB3* is highly upregulated in Pgb+ embryos in the first hours after imbibition, and downregulated at 48 h. This may indicate its particular role in supplying the cytosolic NADH to the mitochondrial ETC upon the depletion of oxygen, and its lower contribution during the aerobic phase.

## 4. Materials and Methods

### 4.1. Plant Growth

The transgenic lines of barley (*Hordeum vulgare* L. var. Golden Promise) seeds with overexpression and knockdown of the Pgb1 (Pgb+, Pgb-) were obtained from Aarhus University, Denmark, where they were constructed as described earlier [53,54]. Single independent transformants and the wild type plants were used for all experiments. Seeds were soaked with sterile deionized water on filter paper in Petri dishes in darkness at 25 °C. To gain insight into the biochemical and molecular changes during germination, an extensive time course was examined, from dry seeds to radicle protrusion (at 15–20 h postimbibition) and up to 48 h. Embryos (usually 100 mg) were isolated and ground in liquid nitrogen using a mortar and pestle for studying gene expression and assaying several metabolic parameters.

### 4.2. ATP/ADP Ratio

Fresh biomass (100 mg) was homogenized with the addition of 1 mL of 2.4 M perchloric acid. The homogenate was then neutralized using 5 M KOH and centrifuged at 16,000× *g* for 10 min at 4 °C [55]. The ADP/ATP ratio was measured by a luciferase-based assay kit (Enzylight TM ADP/ATP ratio assay kit; Bioassay Systems, Hayward, CA, USA), following the manual instructions.

### 4.3. NO Emission

NO emission was measured using a chemiluminescent detector (CLD 88 p; Eco-Physics, Dürnten, Switzerland), as described earlier [34,56], and averaging total NO accumulation every 30 min. The measuring gas was kept NO free using a NO scrubber supplied by Eco Physics Ltd., Switzerland. Gas flow was regulated by flow controllers (Thermo Fisher Scientific, Waltham, MA, USA).

### 4.4. Measurement of Protein Concentration

The total concentration of proteins was measured using Bradford reagent (Sigma–Aldrich, St. Louis, MO, USA) and bovine serum albumin as a standard [57].

### 4.5. Total Protein S-Nitrosylation

The measurement of the protein *S*-nitrosylation was performed following Ma et al. [5]. The method is based on the reduction of R-SNO to R-SH in the presence of ascorbate and detecting free thiol groups by 5,5′-dithiol-bis (2-nitrobenzoic acid) (DTNB). Extraction was achieved using 50 mM HEPES (pH 8.0) containing 1 mM EDTA, 0.1 mM neocuproine, 0.2% (*w*/*v*) SDS and 0.5% (*w*/*v*) CHAPS. The homogenate was centrifuged at 15,000× *g* for 10 min at 4 °C, and the supernatant was incubated overnight in ice-cold acetone (−20 °C). Then, protein precipitate was separated by centrifuging, the subsequent pellet was washed several times with chilled 70% acetone, before being resuspended in the same volume of the extraction buffer. Protein solution was separated into two 0.9 mL samples, adding 50 μL of 100 mM ascorbate to the experimental tube and the same volume of water to the control. After incubating for 1 h at room temperature, 50 μL of 10 mM DTNB in 75 mM potassium phosphate buffer (pH 7.0) was added, and the optical density of both samples was measured at 412 nm. The mixture of ascorbate and DTNB in the extraction buffer and DTNB in the same buffer were set up as a blank for the treatment and control groups, respectively. The difference of R-SH quantity between sample and control groups was taken for calculation of R-SNO level. The quantity of R-SH generated by ascorbate treatment corresponded to that of R-SNO in proteins. The evaluation of free SH-groups in proteins was performed without ascorbate treatment.

### 4.6. Enzymatic Activity Assays

Alcohol dehydrogenase (ADH; EC 1.1.1.1) activity was assayed by measuring the reduction of NAD+ in the course of oxidation of ethanol at 340 nm, as previously described [58]. *S-*Nitrosoglutathione reductase (GSNOR or ADH3; EC 1.2.1.46) activity was assayed spectrophotometrically at 25 °C by monitoring the oxidation of NADH in the presence of *S*-nitrosoglutathione (GSNO) at 340 nm [9].

### 4.7. Gene Expression

The primers of target genes for the quantitative real-time polymerase chain reaction (qRT PCR) were designed using thr NCBI/Primer-BLAST, according to known cDNA sequences of the *Pgb1*, *NR*, *NiR*, *ADH1*, *ADH3*, *SDH-A*, *SDH-B*, *PDC-E1*, *PDC-E2*, *AOX1a*, *AOX1d1*, *NDB2* and *NDB3* genes. They encode correspondingly the class 1 phytoglobin, nitrate reductase (EC 1.7.99.4), nitrite reductase (EC 1.7.2.1), alcohol dehydrogenase (EC 1.1.1.1), GSNOR (or ADH3; EC 1.2.1.46), flavoprotein subunit A and iron–sulfur protein subunit B of succinate dehydrogenase (EC 1.3.5.1), pyruvate dehydrogenase (EC 1.2.4.1) and dihydrolipoyl transacetylase (EC 2.3.1.12) subunits of the pyruvate dehydrogenase complex, two forms of alternative oxidase (EC 1.10.3.11) and two forms of the externally facing rotenone-insensitive NADH dehydrogenase. Glyceraldehyde 3-phosphate dehydrogenase (GAPDH; EC 1.2.1.12) was set up as a reference gene. Specific primers of the target genes are listed in Table 1.

Extraction of total RNA was performed using the FastRNA^®^ Pro Green Kit (MP Biomedicals, Irvine, CA, USA), according to the standard protocol of the manufacturer. Reverse transcription of RNA was conducted according to the protocol for the SuperScript™ II Reverse Transcriptase (Invitrogen, Carlsbad, CA, USA). The single strand cDNA was used as a template in the following PCR. PCR was performed on an Applied Biosystems (Foster City, CA, USA) StepOnePlus Real-Time PCR System. The procedure followed the manufacturer’s protocol for the SYBR Green qPCR Master Mixes (Thermo Fisher Scientific, Waltham, MA, USA): 0.4 µL 10 µM forward primer, 0.4 µL 10 μM reverse primer, 1 µL cDNA and 5 µL SYBR Green qPCR Master Mixes were mixed and adjusted to 3.2 µL using nuclease-free water. Biological replicates corresponded to independent RNA extracts, and three technical replications were run for each biological replicate. The program for the RT PCR reactions was set up as the initial activation at 95°C for 30 s, followed by 40 cycles of 95 °C for 10 s and 61 °C for 45 s; followed by melting curve analysis obtained by heating to 95 °C for 15 s, cooling to 55 °C and incrementally heating to 95 °C at the rate of 0.05 °C s-1. Standard curves of target genes were made based on a 5-fold dilution series for the genomic barley cDNA (*E* = 1.3–1.8, *R*^2^ = 0.93–0.99). The amount of target genes in unknown samples was calculated from the cycle threshold (*C*t) using the standard curve.

### 4.8. Statistical Analysis

All the experiments were repeated at least three times. The statistical analyses were performed using SPSS software (Statistical Package for Social Science; version 21, Chicago, IL, USA). A one-way ANOVA was performed to identify significant differences among different lines of barley (*p* ≤ 0.05) and measured by using Duncan’s multiple range. The data in the text, table and figures are expressed as means ± standard deviations of three replicates. The differences with *p* ≤ 0.05 were considered as statistically significant.

## 5. Conclusions

*Pgb1* expression in embryo is essential for the maintenance of redox and energy balance before radicle protrusion, when seeds experience low internal oxygen concentration. It also exerts the effect on metabolism and gene expression during the initial development of seedlings. Overall, Pgb1, by participating in NO turnover in the Pgb-NO cycle, influences the ability of the embryo to maintain the delicate balance of energy production and utilization, which is of fundamental importance for the growth and development of germinated seeds.

## Figures and Tables

**Table 1:** Primers used for qRT-PCR assays of germinating seeds of barley.

Primer	Forward Sequence	Reverse Sequence	Accession Number
Pgb 1	5′-ACCAACCCCAAGCTCAAGAC-3′	5′-CTGCCACGCCGTATTTCAAG-3′	U94968.1
NR	5′-CAACACCAACTCCGTCAT-3′	5′-CTGAGTATGCGTATCCCTTG-3′	X57844.1
NiR	5′-GACATCGGCTTCATGGGCT-3′	5′-GCACGGCCTTCTTGTACACC-3′	S78730.1
ADH1	5‘-GATCTGCTCAGGATCAACAC-3′	5′-GTGGAAGTCCCTACGAAATG-3′	AF253472.1
ADH3	5′-GTCTCTCAACTGGACTTGGTG-3′	5′-TAGCTTGTTCGTATTTTGCAGG-3′	X12734.1
SDH-A	5′-CAGTGAAGGTGAGCGTTTCA-3′	5′-CACCAGCAAAAATAGCAGCA-3′	AK376855.1
SDH-B	5′-TGTACGAGTGCATCCTCTGC-3′	5′-TCGTCGTTGATGGAGTCAAG-3′	AK372209.1
PDC-E1	5′-ATTGAATTCCGCCCTTGACG-3′	5′-GCCAGTAAAACCAGCCTCTG-3′	AK353615.1
PDC-E2	5′-TGCAGGGATGGAAAGAGCTT-3′	5′-GGTTGGAGCTGCTTCATACG-3′	AK362954.1
AOX1a	5′-CGTCAACCACTTCGCATCGG-3′	5′-GCCCTCATTTCCTCGGAAGC-3′	AK363239.1
AOX1d1	5′-CACTACGCATCCGACATCCA-3′	5′-CAACAATCCATCCAAATTAACG-3′	AK365405.1
NDB2	5′-CGTCCACTGTCGCTCTGC-3′	5′-GGCATCCTCCACTTCCTTCAG-3′	AK367948.1
NDB3	5′-GCAAAATCCAGCTACTGGCG-3′	5′-TTCACGCACCCTTAGCCATT-3′	AK354220.1
GAPDH	5′-GCTCAAGGGTATCATGGGTTACG-3′	5′GCAATTCCACCCTTAGCATCAAAG-3′	AB120301.1

## Abbreviations

ADH	Alcohol dehydrogenase
AOX	Alternative oxidase
DTNB	5,5′-dithiol-bis (2-nitrobenzoic acid)
ETC	Electron transport chain
GAPDH	Glyceraldehyde 3-phosphate dehydrogenase
GSNO	*S*-nitrosoglutathione
GSNOR	*S*-nitrosoglutathione reductase
NDs	type II NAD(P)H dehydrogenases
NiR	Nitrite reductase
NO	Nitric oxide
NR	Nitrate reductase
PDC	Pyruvate dehydrogenase complex
Pgb	Phytoglobin
RNS	Reactive species of nitrogen
ROS	Reactive species of oxygen
SDH	Succinate dehydrogenase
TCA	Tricarboxylic acid
